# Polyethylene Glycolylation of the Purified Basic Protein (Protamine) of Squid (*Symplectoteuthis oualaniensis*): Structural Changes and Evaluation of Proliferative Effects on Fibroblast

**DOI:** 10.3390/ijms26051869

**Published:** 2025-02-21

**Authors:** Na Li, Jiren Xu, Yu Li, Jeevithan Elango, Wenhui Wu

**Affiliations:** 1 Department of Marine Pharmacology, College of Food Science and Technology, Shanghai Ocean University, Shanghai 201306, China; 18335440803@163.com (N.L.); mikumiyo@hotmail.com (J.X.); a965748659@163.com (Y.L.); 2 Department of Biomaterials Engineering, Faculty of Health Sciences, UCAM-Universidad Católica San Antonio de Murcia, Guadalupe, 30107 Murcia, Spain; 3 Center of Molecular Medicine and Diagnostics (COMManD), Department of Biochemistry, Saveetha Dental College and Hospitals, Saveetha Institute of Medical and Technical Sciences, Saveetha University, Chennai 600077, India; 4 Marine Biomedical Science and Technology Innovation Platform of Lin-gang Special Area, Shanghai 201306, China; 5 Putuo Branch of International Combined Research Center for Marine Biological Sciences, Zhoushan 316104, China

**Keywords:** protamine, purification, PEGylation, structural characterization, signaling pathway

## Abstract

In recent years, arginine-rich basic proteins have garnered significant attention due to their essential roles in various biological processes. However, the potential of marine-derived proteins in this domain remains largely unexplored. This study presents, for the first time, the isolation and purification of a 14.3 kDa protamine (SOP) from the mature spermatogonial tissues of *Symplectoteuthis oualaniensis*. Additionally, we obtained an 18.5 kDa PEGylated derivative, SOP-PEG. The physicochemical properties of both SOP and SOP-PEG were comprehensively characterized using SEM, FTIR, CD, and TGA. PEGylation markedly altered the surface morphology, secondary structure, and thermal stability of SOP. In vitro studies demonstrated that PEGylation significantly enhanced the biocompatibility of SOP, leading to improved proliferation of L-929 fibroblasts. Furthermore, both SOP and its PEGylated derivative (SOP-PEG) regulated the cell cycle, activated the PI3K-Akt signaling pathway, and modulated anti-apoptotic mechanisms, suggesting their potential to support cell survival and facilitate tissue regeneration. Notably, SOP-PEG exhibited superior bioactivity, likely attributable to its enhanced delivery efficiency conferred by PEGylation. Collectively, these findings underscore the promising applications of SOP and SOP-PEG in regenerative medicine and highlight the pivotal role of PEGylation in augmenting the bioactivity of SOP.

## 1. Introduction

With the advancement of research on marine biological resources, marine organisms have emerged as a vital source for the development of novel drugs and functional materials, owing to their unique biological environment and abundance of bioactive molecules [1,2,3]. Protamine, a class of small, arginine-rich, basic proteins with molecular weights typically ranging from 5 kDa to 20 kDa, is predominantly found in the sperm cells of certain fish and marine invertebrates [4]. These proteins are characterized by their distinctive molecular structure and remarkable biological activities. Currently, protamine has been successfully extracted from *Coregonus peled* [5], *Clupea harengus* [6] and human sperm [7]. As a small molecular protein, protamine is a natively disordered protein that does not possess a defined 3D structure. It binds to nucleic acids (such as DNA and RNA) primarily through its predominant positively charged arginine residues, thereby regulating cell proliferation, promoting gene expression, and inhibiting apoptosis [8,9]. These biological functions endow protamine with significant potential in biomedical applications, particularly in tissue repair, anti-cancer therapies, and gene therapy [10,11,12].

The squid (*Symplectoteuthis oualaniensis*) is a rich marine resource that inhabits the deep sea, an extreme environment characterized by high pressure and low oxygen levels. Compared to protamine extracted from mammals or freshwater fish, it exhibits significant growth and reproductive advantages, providing a sustainable source of protamine [13]. These advantages position *Symplectoteuthis oualaniensis* (*S. oualaniensis*) protamine as a promising raw material for biomedical development. However, studies on the isolation, purification, and structural characterization of protamine from *S. oualaniensis* remain limited, and its regulatory effects on cellular functions are rarely reported. L-929 cells, widely used as a fibroblast model in cell biology research, are highly sensitive to external stimuli, making them an ideal system for studying the bioactivity of biomolecules [14,15,16]. The regulation of L-929 cell proliferation and function is of great significance in the fields of tissue repair and regenerative medicine.

In recent years, polyethylene glycol (PEG) modification technology has been extensively studied for its applications in protein engineering and drug delivery [17,18,19]. PEGylation enhances the solubility, stability, and half-life of proteins by altering their structure and physicochemical properties, thereby improving their biological activity and pharmacological efficacy. Particularly in the development of therapeutic protein drugs, PEGylation has become a widely adopted strategy [20,21,22]. While the performance enhancements of various proteins through PEGylation have been extensively studied, research on *S. oualaniensis* protamine and its PEG-modified derivative remains limited. Systematic investigations into their effects on cellular proliferation and signaling pathway regulation are notably scarce. Therefore, exploring the impact of PEGylation on the biological functions of protamine not only deepens our understanding of its bioactivity but also offers new perspectives for its potential applications in the biomedical field.

Based on this, the present study focuses on *S. oualaniensis* protamine (SOP). Using isolation and purification techniques, a single-component SOP was obtained, followed by structural characterization of SOP and its PEG-modified derivative (SOP-PEG). The study evaluated the impact of PEGylation on the bioactivity of SOP and explored its potential applications in cell proliferation and tissue repair. We specifically investigated the effects of SOP and SOP-PEG on L-929 cells, with a particular emphasis on their roles in cell cycle regulation, activation of the PI3K-Akt signaling pathway, and modulation of anti-apoptotic pathways.

## 2. Results

### 2.1. Isolation and Purification of SOP with Sakaguchi Reaction

**Figure 1:**
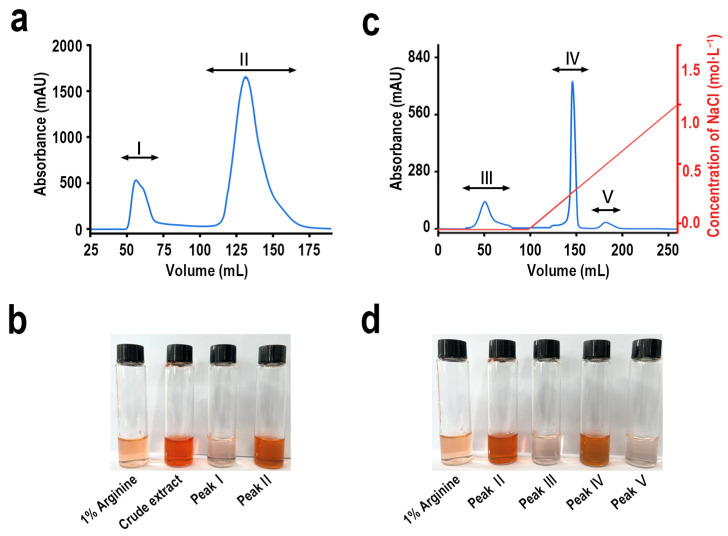
Separation and purification of protamine from *S. oualaniensis*. (**a**) Gel filtration chromatography of crude protamine extract using a Superdex G-50 column. (**b**) Sakaguchi reaction: show 1% arginine, crude protamine extract, fraction I, and fraction III, respectively. (**c**) Ion-exchange chromatography of fraction II using CM Sepharose Fast Flow. (**d**) Sakaguchi reaction: show fractions 1% arginine, fraction II, fraction III, fraction IV and fraction V, respectively.

The protamine from the testicles of *S. oualaniensis* was isolated and purified using the process described below. Protamine was monitored using the Sakaguchi reaction as an indicator to assess the components obtained from each step of the isolation and purification [23]. The crude protamine extract was dissolved in distilled water, filtered through a 0.45 µm membrane, and then separated into two fractions (I and III) using a Sephadex G-50 column (Figure 1a). Based on the Sakaguchi reaction, a distinct color reaction was observed in fraction II (Figure 1b). Fraction II was then dialyzed, freeze-dried, and further purified using a CM Sepharose Fast Flow ion exchange column. The second peak (fraction IV in Figure 1c exhibited a clear color reaction (Figure 1d), indicating that fraction ii was the target product.

### 2.2. SDS-PAGE Analysis of SOP and SOP-PEG

**Figure 2:**
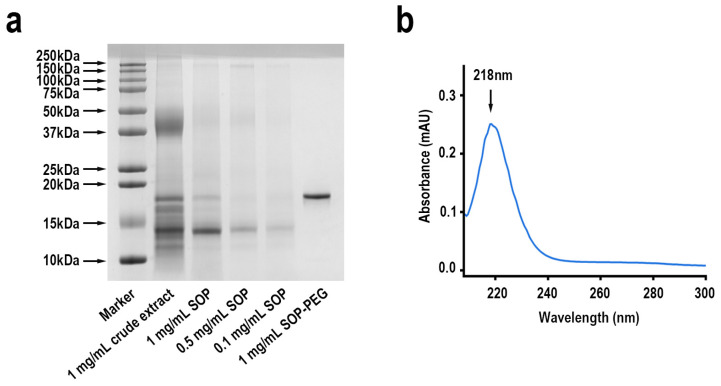
(**a**) SDS-PAGE separation (15%) of crude extract and purified protamine, stained with Coomassie Brilliant Blue R-250, followed by transfer to decolorization solution (Vethanol:Vacetic acid:VH_2_O = 2:1:7). Lane 1: molecular weight markers (10–250 kDa); Lane 2: crude protamine extract (1 mg/mL); Lane 3: SOP (1 mg/mL); Lane 4: SOP (0.5 mg/mL); Lane 5: SOP (0.1 mg/mL); Lane 6: SOP-PEG (1 mg/mL). (**b**) UV–Vis spectrum of SOP.

The SDS-PAGE results demonstrated that the purified protamine from *S. oualaniensis* produced a major band, designated as SOP (*S. oualaniensis* protamine) (Figure 2a and Figure S1), along with other minor bands. By comparing the relative migration rate of SOP with molecular weight markers, its molecular weight was determined to be 14.3 kDa. It is noteworthy that the molecular weight of SOP-PEG increased to 18.5 kDa, with the PEG having a molecular weight of 2000 Da. This result indicates that successful PEGylation of SOP occurred, with approximately two PEG molecules attached to each SOP molecule. According to previous studies, protamine is a small protein with molecular weights ranging from 5 kDa to 20 kDa [6,24,25], placing SOP within this expected range. As anticipated, the bandwidth and intensity increased with rising protein concentrations (from 0.1 mg/mL to 1 mg/mL). Furthermore, SOP exhibited a single ultraviolet absorption peak at 218 nm (Figure 2b).

### 2.3. Amino Acid Composition of SOP

Protamine, particularly derived from *S. oualaniensis* (SOP), exhibits a significantly higher content of basic amino acids compared to other proteins [26,27,28]. The amino acid composition analysis reveals that SOP is rich in basic amino acids, particularly arginine (Arg) and lysine (Lys), which are characteristic of protamine (Table 1) [29]. These amino acids account for approximately 418.1 residues per 1000 residues, a proportion consistent with the highly basic nature of protamine. Specifically, these basic amino acids form electrostatic interactions with the phosphate backbone of DNA, suggesting that SOP may possess strong DNA-binding capabilities [30,31,32]. Additionally, when compared to protamine from other species, SOP has a relatively higher proportion of hydrophobic amino acids, such as leucine and isoleucine. This observation indicates that these hydrophobic residues might play crucial roles in maintaining protein stability or in transmembrane processes [33,34]. Notably, the elevated levels of arginine in SOP may enhance its function in chromatin condensation and gene regulation, indicating its potential involvement in nucleic acid-related activities [35,36]. Furthermore, the balanced distribution of cysteine (Cys) in SOP suggests its possible antioxidant properties, which may offer protection against oxidative damage in cells [37,38]. These unique features underscore the close relationship between the amino acid composition of SOP and its biological activity. This foundational understanding paves the way for further investigation into SOP’s potential in applications such as skin repair, anti-tumor therapy, and immune modulation.

### 2.4. Microstructure Analysis of SOP and SOP-PEG

**Figure 3:**
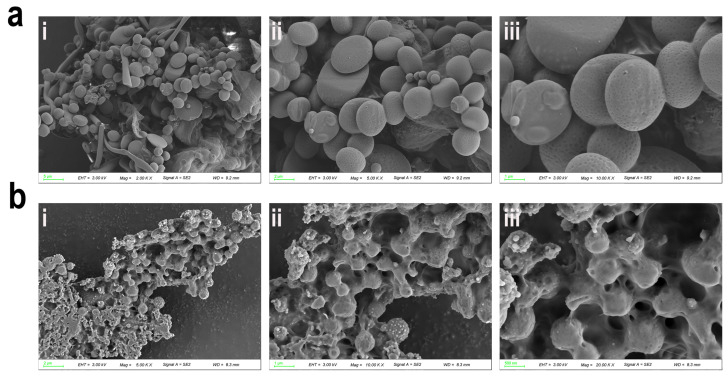
Microstructural analysis of SOP (**a**) (i: 5 μm; ii: 2 μm; iii: 1 μm) and SOP-PEG (**b**) (i: 2 μm; ii: 1 μm; iii: 500 nm): scanning electron microscopy (SEM) images at different magnifications.

We further investigated the microstructural characteristics of SOP and SOP-PEG using scanning electron microscopy (SEM) at various magnifications. The results revealed that SOP displayed a smooth, oval-shaped structure with a uniform surface and no significant attachments, indicating a high degree of homogeneity and stability in its native form (Figure 3a). In contrast, following PEGylation, SOP-PEG exhibited a roughened surface with distinct encapsulating features (Figure 3b). This structural change suggests that PEG molecules have been successfully conjugated or cross-linked to the surface of the protamine. The observed surface roughness indicates that PEGylation alters the protein’s surface properties, enhancing its stability and resistance to degradation in complex biological environments [40,41]. Moreover, the roughened morphology may facilitate more efficient interactions with target cells or biological factors, potentially improving its biological activity [42,43]. This structural modification provides a solid foundation for further studies into the functional properties of SOP-PEG. Compared to the unmodified SOP, the PEGylated SOP may exhibit significant advantages in terms of biological activity. In conclusion, PEG modification not only markedly changes the surface morphology of protamine but also imparts enhanced potential for optimized biological functionality, offering substantial research and application value.

### 2.5. Secondary Structure Analysis of SOP and SOP-PEG

**Figure 4:**
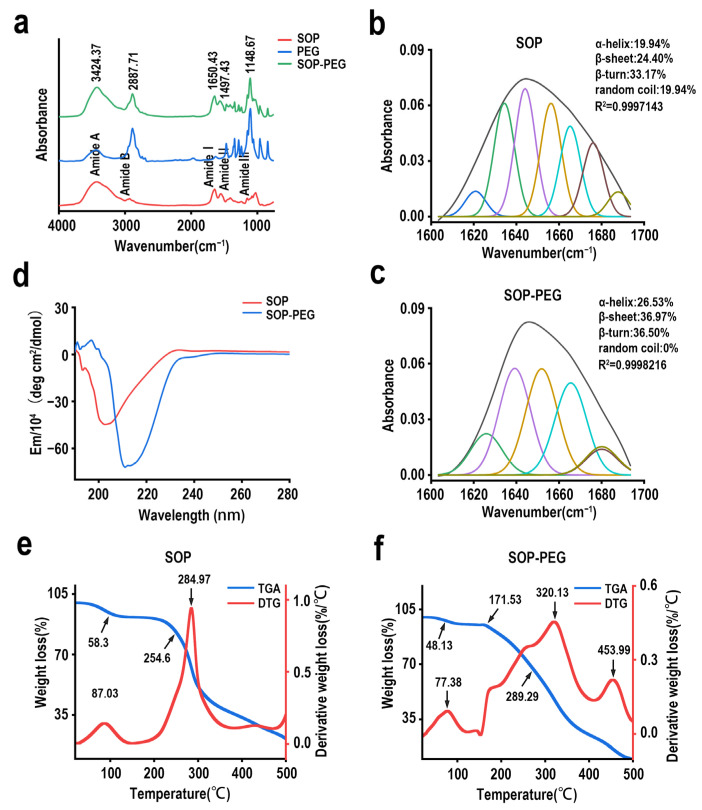
(**a**) Fourier-transform infrared (FTIR) spectra of SOP and SOP-PEG; (**b**,**c**) deconvolution analysis of the secondary structure of SOP and SOP-PEG based on the amide I band (1600–1700 cm^−1^); (**d**) circular dichroism spectrum of SOP and SOP-PEG; (**e**,**f**) thermogravimetric analysis (TGA) and derivative thermogravimetric analysis (DTG) curves of SOP and SOP-PEG.

SOP, as a natural protein, exhibits favorable biological activity, while PEG modification of proteins significantly enhances their stability, efficacy, and safety, making it a preferred modification strategy for many protein-based drug formulations [44,45,46]. The results shown in Figure 4a indicate a noticeable enhancement in signals for the PEG-modified protamine at 3400–3500 cm^−1^ (–OH stretching vibration) and 1100–1140 cm^−1^ (C–O–C stretching vibration). In the amide I and II bands, slight shifts or changes in intensity were observed following PEG modification. Notably, a new peak, attributed to the C–O–C vibration of PEG, appeared within the 1000–1200 cm^−1^ range, further confirming the successful attachment of PEG molecules to the protamine. The secondary structure analysis of SOP and SOP-PEG through CD and infrared spectroscopy reveals significant differences. The CD results indicate that SOP predominantly consists of random coils (51.8%), whereas the PEG-modified SOP-PEG exhibits a higher content of α-helix (53.1%) and β-sheet (46.9%), with a near-complete disappearance of random coils (Figure 4d, Table 2). This suggests that PEG modification enhances the ordered structure of SOP. Additionally, infrared spectroscopy results also show an increase in α-helix and β-sheet content in SOP-PEG, with a reduction in random coils (Figure 4b,c). These findings highlight that PEG modification has a substantial impact on the secondary structure of SOP, promoting structural stability and order.

### 2.6. Thermal Stability Analysis of SOP and SOP-PEG

The secondary structure analysis revealed that both SOP and SOP-PEG maintain favorable structural characteristics, which are crucial for their stability. TGA and DTG results demonstrate that PEG modification significantly alters the thermal decomposition behavior of SOP and enhances its thermal stability (Figure 4e,f). The TGA curve of SOP shows two characteristic points at 58.3 °C and 254.6 °C, corresponding to the evaporation of adsorbed water and the thermal decomposition of the protein backbone, respectively. The DTG peaks of SOP were observed at 87.03 °C and 284.97 °C. In contrast, the TGA curve of SOP-PEG introduces an additional decomposition stage at 171.53 °C, with characteristic points at 48.13 °C, 171.53 °C, and 269.29 °C. The DTG peaks of SOP-PEG occur at 77.38 °C, 320.13 °C, and 453.99 °C. PEG modification introduces a new thermal decomposition behavior at 171.53 °C, significantly raises the backbone decomposition temperature (289.29 °C), and enhances the oxidation decomposition temperature of residual carbon (453.99 °C). These results suggest that PEG provides protective effects to the protein, thereby improving its thermal stability. This optimization effect offers potential for protein-based biomaterials in complex environments and highlights the important role of PEG in regulating protein degradation mechanisms.

### 2.7. Cell Viability Assessment

**Figure 5:**
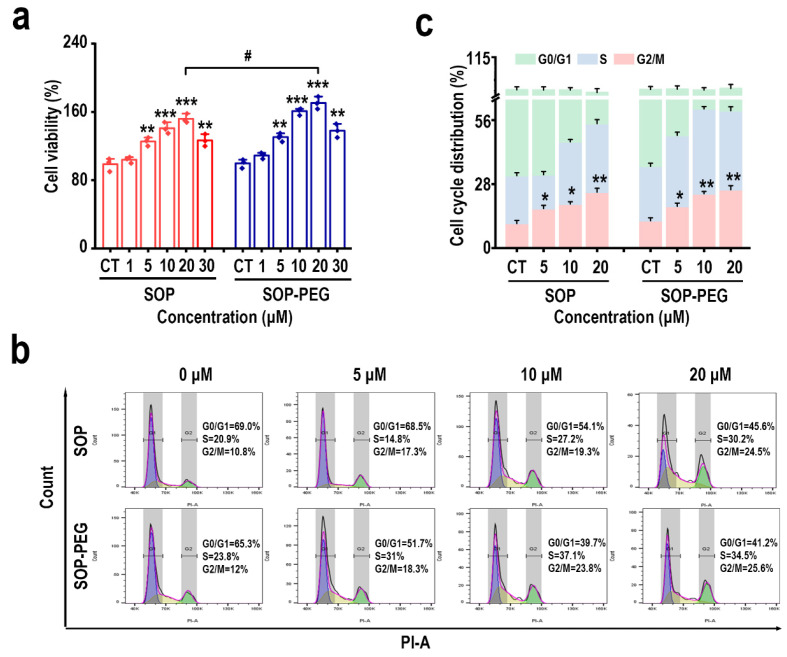
Effects of protamine on the proliferation and cell cycle regulation of L-929 cells. (**a**) Influence of SOP and SOP-PEG on L-929 cell proliferation. (**b**) Regulation of cell cycle progression in RAW264.7 cells by SOP and SOP-PEG. (**c**) Statistical analysis of the cell cycle distribution after treatment with SOP and SOP-PEG. * *p* < 0.05, ** *p* < 0.01, and *** *p* < 0.001 indicate significant differences compared to the control group. # indicates a significant difference between the two treatment groups (*p* < 0.05).

To investigate the effects of SOP and SOP-PEG on L-929 cell proliferation, L-929 cells were co-incubated with varying concentrations of SOP and SOP-PEG for 48 h. The results of the CCK-8 assay are shown in Figure 5a. The data indicate that, compared to the control group (PBS), the proliferation of L-929 cells increased with the increasing concentrations of both SOP and SOP-PEG. Notably, the proliferation of L-929 cells increased with concentrations of SOP and SOP-PEG ranging from 1 to 20 μM; however, a decline in cell proliferation was observed at 30 μM. It is worth mentioning that SOP-PEG exhibited a more pronounced proliferative effect than SOP, with significant differences observed at 20 μM. In conclusion, both SOP and SOP-PEG demonstrated positive bioactivity in promoting L-929 cell proliferation, with PEG modification further enhancing the proliferative effect of SOP on L-929 cells.

### 2.8. Effects of SOP and SOP-PEG on the Cell Cycle of L-929 Cells

The proliferation of L-929 cells is tightly regulated through the cell cycle, with the G2/M phase playing a critical role in the completion of DNA replication and the preparation for cell division [48,49,50]. Flow cytometry analysis (Figure 5b,c) revealed that after treatment with protamine (SOP and SOP-PEG), the proportion of L-929 cells in the G2/M phase was significantly higher compared to the control group. This suggests that protamine may promote the progression of the cell cycle from the G2 to the M phase, thereby enhancing cell division and ultimately increasing the proliferative capacity of the cells.

### 2.9. Effects of SOP and SOP-PEG on G2/M Phase-Related Genes and Proteins in L-929 Cells

**Figure 6:**
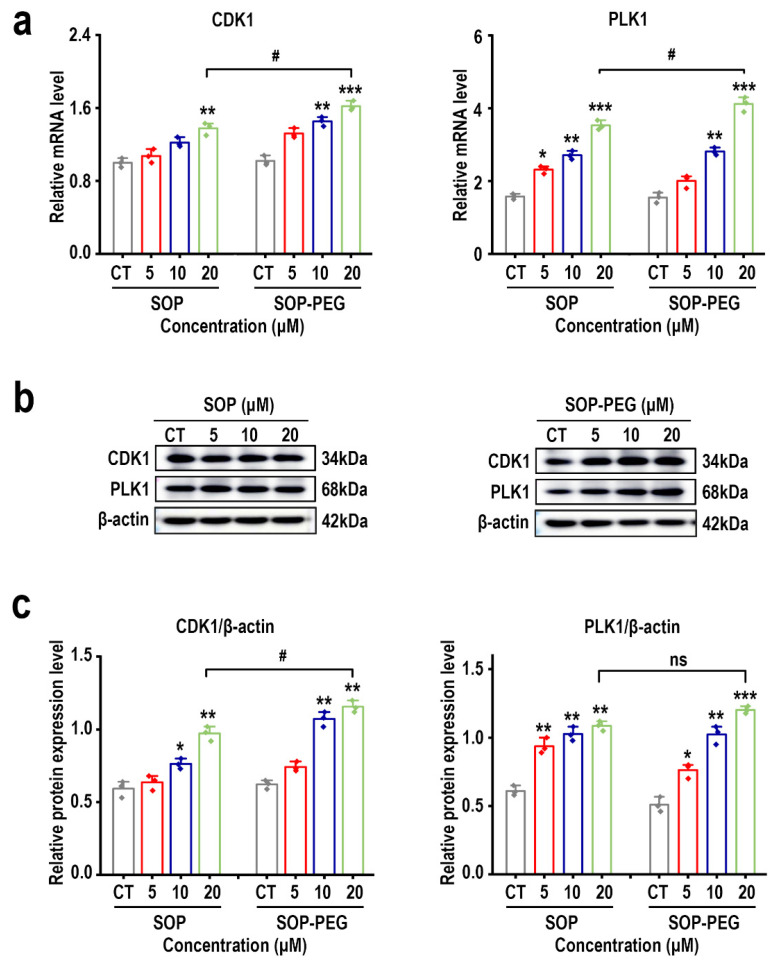
(**a**) The effect of SOP and SOP-PEG on the mRNA expression of CDK1 and PLK1 in L-929 cells. (**b**) The effect of SOP and SOP-PEG on the protein expression levels of G2/M phase-related proteins in L-929 cells. (**c**) Statistical analysis of CDK1 and PLK1 protein expression, with β-actin used as an endogenous loading control. * *p* < 0.05, ** *p* < 0.01, and *** *p* < 0.001 indicate significant differences compared to the control group. # indicates a significant difference between the two treatment groups (*p* < 0.05), and ns indicates no significant difference.

*CDK1* (Cyclin-Dependent Kinase 1) and *PLK1* (Polo-Like Kinase 1) are two key protein kinases involved in cell cycle regulation, working synergistically to drive the transition from G2 to M phase and the initiation of mitosis [51,52,53]. qRT-PCR analysis revealed that, compared to the control group, the mRNA expression levels of *CDK1* and *PLK1* were significantly upregulated in both the SOP and SOP-PEG treatment groups (Figure 6a). Furthermore, Western blot (WB) analysis confirmed this upregulation, with CDK1 and PLK1 protein levels also significantly increased following treatment, aligning with the mRNA expression trends (Figure 6b,c and Figure S2). Notably, SOP-PEG exhibited a more pronounced effect on cell cycle promotion. The qRT-PCR results showed that the mRNA expression levels of *CDK1* and *PLK1* were significantly higher in the SOP-PEG treatment group compared to the SOP group. Corresponding WB results also demonstrated a substantial increase in protein expression. These findings suggest that PEG modification not only preserves the biological activity of SOP but also enhances its stability and cellular uptake efficiency, thereby further amplifying its regulatory effect on the cell cycle.

### 2.10. SOP and SOP-PEG Effects on PI3K-Akt Signaling Pathway-Related Genes and Proteins in L-929 Cells

**Figure 7:**
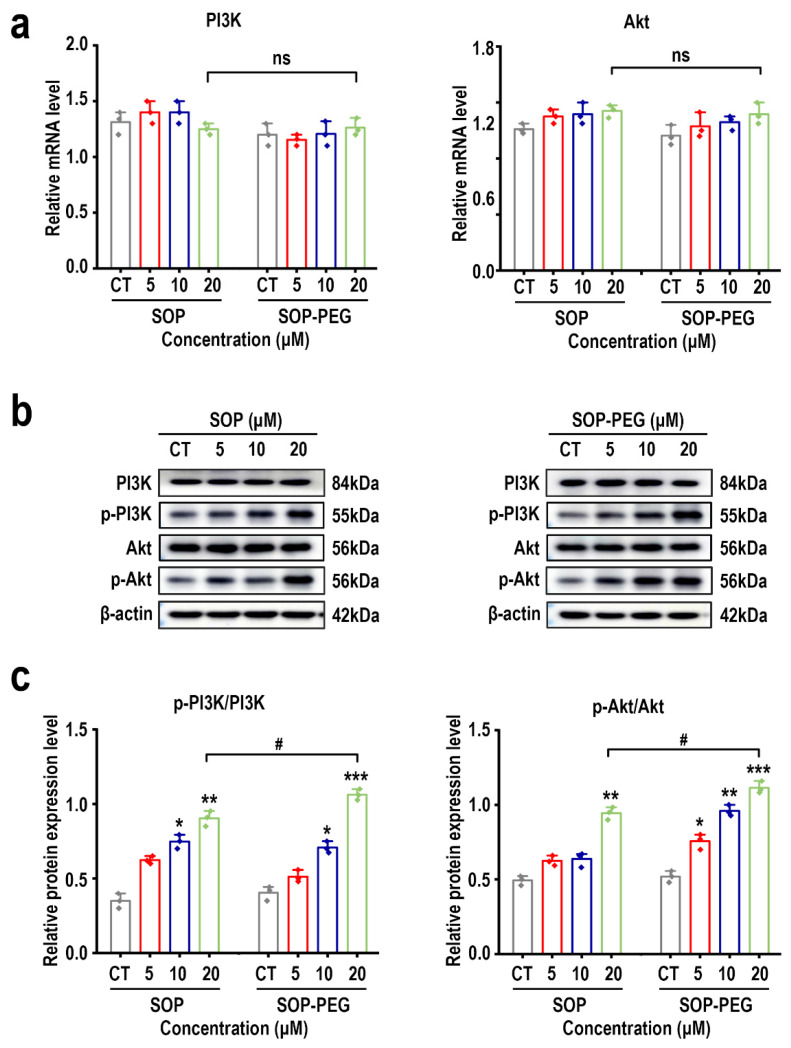
Effects of SOP and SOP-PEG on the PI3K-Akt signaling pathway in L-929 cells. (**a**) Effects of SOP and SOP-PEG on the mRNA expression of PI3K and Akt in L-929 cells. (**b**) Effects of SOP and SOP-PEG on the protein expression levels of PI3K-Akt signaling pathway-related proteins in L-929 cells. (**c**) Statistical analysis of p-PI3K/PI3K and p-Akt/Akt protein expression levels, with β-actin as an internal loading control. *, **, and *** represent *p* < 0.05, *p* < 0.01, and *p* < 0.001 compared to the control group, respectively. # indicates a significant difference between the two groups (*p* < 0.05), and ns denotes no significant difference.

The PI3K-Akt signaling pathway plays a critical role in various biological processes, including cell proliferation, survival, and growth [54,55,56]. We first performed RT-PCR analysis to evaluate the mRNA expression levels of *PI3K* and *Akt*. Surprisingly, neither SOP nor SOP-PEG significantly altered the mRNA expression levels of *PI3K* and *Akt* compared to the control group, with no statistically significant differences observed (Figure 7a). This result suggests that, although SOP and SOP-PEG can affect cell proliferation, their effects are not mediated by changes in the transcriptional levels of *PI3K* and *Akt* genes. Next, we analyzed the phosphorylation states of PI3K and Akt through Western blot to explore their involvement in signal transduction. The results showed that both SOP and SOP-PEG significantly enhanced the phosphorylation levels of PI3K and Akt (Figure 7b,c and Figure S3). Notably, PEG-modified SOP exhibited a more pronounced effect on promoting the phosphorylation of PI3K and Akt. This indicates that, despite no significant differences at the transcriptional level, SOP and SOP-PEG can activate the PI3K-Akt signaling pathway through post-transcriptional regulation, thereby promoting the proliferation of L-929 cells.

### 2.11. Effects of SOP and SOP-PEG on Apoptosis-Related Genes and Proteins in L-929 Cells

**Figure 8:**
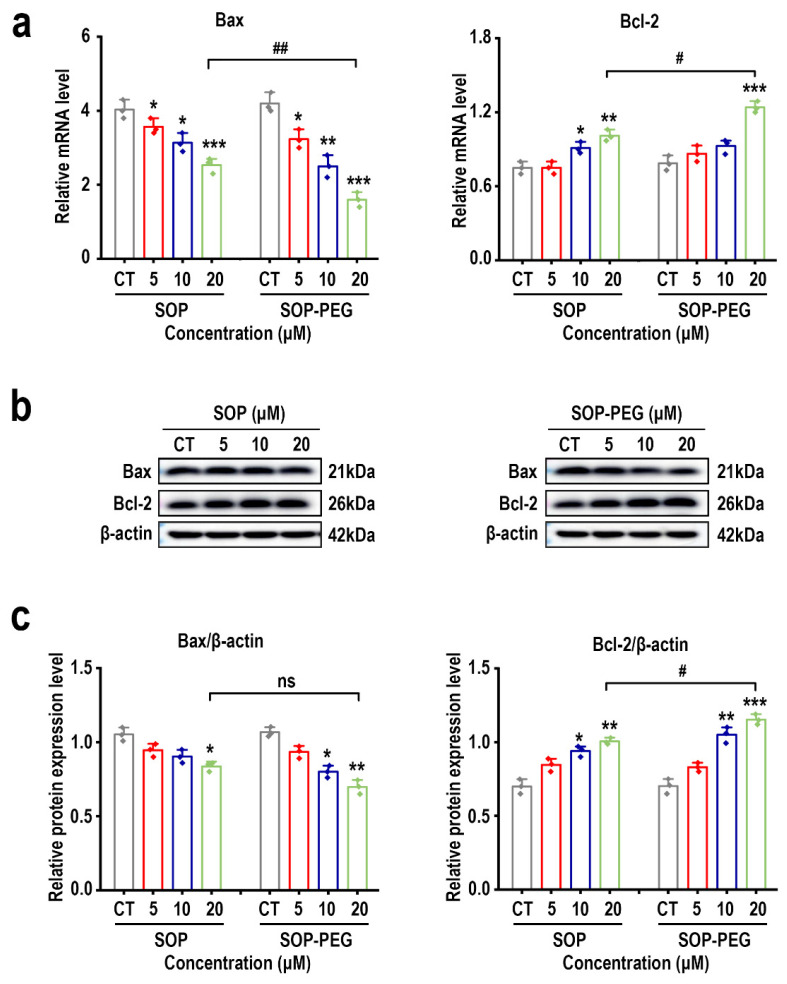
(**a**) Effects of SOP and SOP-PEG on the mRNA expression of Bax and Bcl-2 in L-929 cells. (**b**) Effects of SOP and SOP-PEG on the expression levels of apoptosis-related proteins in L-929 cells. (**c**) Statistical results of Bax and Bcl-2 protein expression, with β-actin as the endogenous loading control. *, **, and *** indicate *p*-values < 0.05, 0.01, and 0.001, respectively, compared to the control group. # and ## represent significant differences between the two groups at *p* < 0.05 and *p* < 0.01, respectively. ns indicates no significant difference.

The PI3K-Akt pathway plays a key role in both cell proliferation and apoptosis inhibition [57]. Akt enhances cell survival by phosphorylating and activating anti-apoptotic proteins while inhibiting pro-apoptotic proteins [58,59]. As expected, both SOP and SOP-PEG significantly inhibited the mRNA expression of the pro-apoptotic gene *Bax* and promoted the mRNA expression of the anti-apoptotic gene *Bcl-2* (Figure 8a). Further Western blot analysis confirmed these findings, showing that SOP and SOP-PEG decreased the expression of Bax protein and increased the levels of Bcl-2 protein (Figure 8b,c and Figure S4). Notably, PEG-modified SOP exhibited a more pronounced effect in regulating the expression of Bax and Bcl-2 proteins, suggesting that PEG modification may enhance SOP’s biological activity or cellular uptake, thereby promoting cell survival and anti-apoptotic ability.

## 3. Discussion

Protamine is a valuable biomolecule, and *S. oualaniensis* represents an abundant marine resource with advantages such as low extraction costs, ample raw material supply, and strong sustainability [60]. In this study, an arginine-rich protein was isolated and purified from the spermary tissue of *S. oualaniensis* and designated as SOP. The purification involved two chromatographic steps using Sephadex G50 Gel filtration chromatography column and CM Sepharose Fast Flow columns, yielding a single protein band, as shown in Figure 1. SDS-PAGE analysis determined the molecular weight of SOP to be 14.3 kDa, consistent with the typical range of protamine (Figure 2a) [6]. Furthermore, the molecular weight of SOP-PEG increased to 18.5 kDa, indicating the successful PEGylation of SOP, with approximately two PEG molecules conjugated per SOP molecule. This modification is likely achieved through covalent bonding, leading to a reduction in SOP’s hydrophobicity while enhancing its solubility and stability. Additionally, PEGylation may influence the conformation and biological function of SOP, potentially improving its in vivo stability and biocompatibility [61,62]. These findings provide a strong foundation for the further application of SOP in biomedical research and therapeutic development. SOP exhibited a distinct absorption peak at 218 nm, characteristic of the ultraviolet absorption of proteins (Figure 2b). This peak primarily originates from the π→π* transition of peptide bonds, indicating the high purity of SOP.

Amino acid composition analysis revealed that SOP is a protamine rich in basic amino acids (Table 3). Notably, SOP contains three basic amino acids—arginine, histidine, and lysine—and is classified as triprotamine. Basic amino acids, such as lysine, arginine, and histidine, are capable of accepting protons through their side chains, resulting in positively charged amino groups [31] (e.g., NH_3_^+^, C (NH_2_)_2_^+^). This leads to the formation of uniformly distributed positively charged regions on the protein surface, which supports its critical role in nucleic acid binding and regulation. Considering both the structural characteristics and functional analysis, it is suggested that SOP may exert its efficient biological functions during cell proliferation and anti-apoptotic processes through its unique structural features. These findings provide a foundation for further mechanistic studies of SOP’s biological activities.

Polyethylene glycol (PEG) modification is a widely used method for protein modification and has been shown to offer significant advantages in various fields [40,41,46]. For instance, PEGylation can substantially enhance protein solubility and stability, effectively reduce protein immunogenicity, significantly extend the protein’s half-life in circulation, and improve its resistance to degradation [63,64,65]. Infrared (IR) spectroscopy analysis demonstrated that PEG modification significantly impacts the protein structure (Figure 4a). In the IR spectrum of SOP, strong absorption peaks corresponding to N-H stretching vibrations (3200–3400 cm^−1^) and C=O stretching vibrations (1650 cm^−1^) indicate that the protein possesses a stable secondary structure [66,67]. After PEG modification, these absorption peaks showed slight shifts, and new characteristic absorption peaks appeared, confirming the successful incorporation of PEG molecules. Both CD and FTIR spectroscopy analyses indicate that PEGylation significantly influences the secondary structure of SOP, though discrepancies exist between the two fitting results. CD analysis suggests that SOP primarily adopts a random coil conformation, whereas PEGylation leads to a substantial increase in α-helix and β-sheet content (Figure 4d and Table 2). In contrast, FTIR spectroscopy reveals that SOP already contains a relatively high proportion of β-sheets, which further increase along with β-turns after PEGylation (Figure 4b,c). These differences likely arise from the varying sensitivities of the two techniques to different secondary structure elements. CD primarily reflects the conformation of proteins in solution, whereas FTIR is more sensitive to hydrogen bonding networks. Thus, integrating data from both methods provides a more comprehensive understanding of the conformational changes induced by PEGylation. PEGylation might also enhance the solubility and stability of the protein by altering its surface polarity and charge, thereby reducing immunogenicity and improving its biological activity and application performance. In conclusion, both FTIR and CD results provide critical evidence for the structural changes induced by PEGylation of protamine.

Thermogravimetric analysis (TGA) and differential thermogravimetric (DTG) analysis further demonstrated that PEG modification significantly altered the thermal decomposition behavior of protamine (SOP) and enhanced its thermal stability (Figure 4e,f). The decomposition of SOP occurs in two primary stages: the evaporation of adsorbed water at lower temperatures and the thermal degradation of the protein backbone. In contrast, SOP-PEG introduced an additional intermediate decomposition stage (171.53 °C), corresponding to the degradation of the PEG segments. Compared to SOP, SOP-PEG showed significantly higher decomposition temperatures for the protein backbone (TGA 269.29 °C, DTG 320.13 °C) and the high-temperature oxidation decomposition temperature (DTG 453.99 °C), suggesting that PEG modification enhances the thermal stability of the protein backbone through intermolecular interactions and improves its resistance to high-temperature oxidation. Additionally, PEG modification resulted in an earlier evaporation temperature for the adsorbed water, reflecting the influence of its hydrophilicity on the material’s properties. Olins et al. suggested that the thermal stability of protamine, typically observed in DNA-polyarginine complexes, indicates the presence of homologous structures in these complexes. They also proposed that protamine molecules exhibit cooperative binding (i.e., non-random assortment) onto the DNA [68]. In conclusion, PEGylation effectively enhances the stability of protamine, making it particularly promising for applications in biopharmaceuticals. Scanning electron microscopy (SEM) results also suggest that PEG molecules may be evenly attached or cross-linked to the surface of protamine, potentially forming a hydrophilic protective layer that enhances its biocompatibility and bioactivity (Figure 3).

Protamine is rich in arginine, which plays a critical role in skin repair [27,69]. In this study, the structural characterization of protamine and its PEGylated derivative revealed that PEG modification improved the protein’s stability and biocompatibility. This structural change may have enhanced the biological activity of protamine in L-929 cells, promoting cell proliferation and thus supporting skin repair. Our findings demonstrated that both SOP and its PEG derivative promoted L-929 cell proliferation and significantly increased the proportion of cells in the G2/M phase (Figure 5). Notably, SOP-PEG exhibited a stronger effect in regulating the cell cycle. mRNA and protein level analysis revealed that SOP-PEG significantly upregulated the expression of *CDK1* and *PLK1*, key molecules involved in the regulation of the G2/M transition (Figure 6). Compared to SOP, PEGylation enhanced the proliferative effect of protamine on cells, potentially by accelerating cell cycle progression and enhancing repair potential.

Furthermore, we observed that both SOP and SOP-PEG were capable of activating the PI3K-Akt signaling pathway, as evidenced by increased phosphorylation of PI3K and Akt-related proteins, though no significant changes were observed at the mRNA level for *PI3K* and *Akt* (Figure 7). This suggests that SOP and SOP-PEG may activate the signaling pathway through post-transcriptional regulation or protein modification mechanisms, rather than through alterations in gene transcription. Additionally, both SOP and SOP-PEG were found to activate anti-apoptotic signaling pathways, as indicated by the inhibition of Bax and the upregulation of Bcl-2, a result that was confirmed at both the mRNA and protein levels (Figure 8). This indicates that SOP and SOP-PEG may effectively delay cell apoptosis and promote cell survival by modulating anti-apoptotic proteins. Notably, PEG modification further enhanced this biological activity, potentially by improving protein stability or intracellular delivery efficiency.

## 4. Materials and Methods

### 4.1. Materials and Reagents

Sodium chloride, sulfuric acid, 95% ethanol, acetic acid-sodium acetate, potassium dihydrogen phosphate-sodium hydroxide, arginine, α-naphthol, hypochlorite, and PEG2000 were purchased from Sinopharm Chemical Reagent Co., Ltd. (Shanghai, China). The Omni-Easy™ One-Step PAGE Gel Preparation Kit (12.5%, catalog number PG213) was obtained from Epizyme Biomedical Technology Co., Ltd. (Shanghai, China). Sephadex G-50 and FF filler were purchased from SolarBio Science & Technology Co., Ltd. (Beijing, China). Coomassie Brilliant Blue R-250 was sourced from Sigma-Aldrich Corporation (St. Louis, MO, USA). The 4× Laemmli sample buffer (catalog number 1610747) and Precision Plus Protein Dual Color Standards (10–250 kDa, catalog number 1610374) were purchased from Bio-Rad Laboratories Inc. (Hercules, CA, USA). The BCA Protein Quantification Kit (catalog number PA115-01) was supplied by Tianjin Biotechnology Co., Ltd. (Beijing, China). Unless otherwise specified, all reagents used were of analytical grade.

### 4.2. Preparation of Crude Protamine Extract from the Testicular Tissue of S. oualaniensis

**Figure 9:**
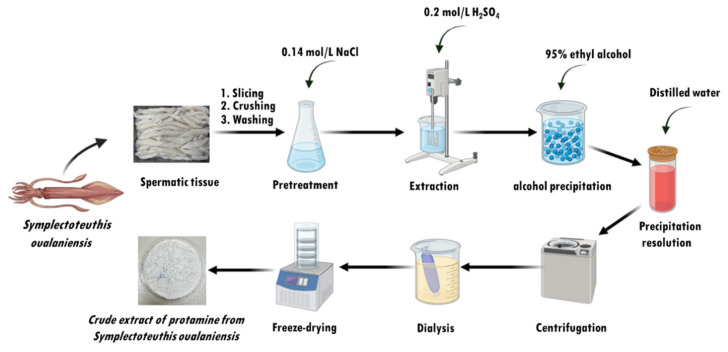
Schematic diagram of the steps involved in protamine extraction from *S. oualaniensis* testicular tissue.

The testicular tissues of *S. oualaniensis* were obtained from the Zhoushan Seafood Market. The extraction of protamine from the testicular tissue was based on the method described by Gill et al. [6], with modifications. All procedures were conducted at 4 °C to ensure sample stability. In brief, 100 g of testicular tissue was immersed in 500 mL of 0.14 mol/L NaCl buffer. The tissue was homogenized three times, each for 1 min, and then stirred for 20 min using an electric stirrer (HUXI, Shanghai, China). The homogenate was then centrifuged at 9900× *g* for 10 min using a Himac CR 21G high-speed floor centrifuge (Hitachi, Tokyo, Japan). The pellet was resuspended in 500 mL of 0.2 mol/L H_2_SO_4_ and stirred for 3 h to extract the protamine. The mixture was then centrifuged at 15,400× *g* for 30 min, and the supernatant was collected. Three volumes of 95% cold ethanol were added to the supernatant to precipitate the proteins. After centrifugation, the precipitate was resuspended in distilled water, and the suspension was dialyzed using dialysis membranes with a molecular weight cutoff (MWCO: 1 kDa) for 48 to 72 h. Following dialysis, the sample was pre-frozen at −80 °C for 8 h and then lyophilized using a freeze-dryer (Labconco FreeZone 2.5 L, Kansas City, MO, USA). A detailed flowchart illustrating the protamine extraction process from *S. oualaniensis* testicular tissue is shown in Figure 9.

### 4.3. Purification of Protamine from S. oualaniensis

Purification was carried out using an AKTA purification system (Inscinstech, Suzhou, China). The crude protamine extract (100 mg) was applied to a Sephadex G50 Gel filtration chromatography column (diameter: 1.6 cm, height: 70 cm) and equilibrated with 25 mM HAC-NaAC buffer (pH 5.4) at a flow rate of 0.5 mL/min. Fractions were collected every 10 mL and monitored using a UV detector at 220 nm. The components were analyzed for color formation using the Sakaguchi reaction. The target fractions were pooled and further purified using a CM Sepharose Fast Flow column (diameter: 2.6 cm, height: 30 cm), equilibrated with 50 mM Gly-NaOH buffer (pH 8.0). The bound proteins were eluted with a 0–1.2 M NaCl gradient in the same buffer at a flow rate of 1 mL/min, collecting every 10 mL. Fractions containing high arginine content were collected and lyophilized to obtain the purified *S. oualaniensis protamine* (SOP).

### 4.4. Sakaguchi Reaction of Protamine

Liu’s method was referenced and modified as follows [39]. A clean test tube was taken, and 10% NaOH solution, 0.2% α-naphthol solution, and sodium hypochlorite solution were sequentially added to the sample in a ratio of 5:1:1:10. The mixture was thoroughly mixed. Simultaneously, a 0.1% arginine solution was prepared as a positive control. The color changes in the solution were observed. Solutions containing protamine exhibited a color change from colorless to red. The absorbance maximum (Amax) of the Sakaguchi reaction is approximately 220 nm.

### 4.5. SDS-PAGE

The molecular size of SOP was determined using SDS-PAGE (15% separating gel and 4.5% stacking gel) according to Laemmli’s method [70]. Different concentrations of SOP solution (1.25, 0.625, and 0.125 mg/mL) were mixed with 5× SDS-PAGE loading buffer (reduced) at a 4:1 (*v*/*v*) ratio. Different concentrations of sample solutions (1, 0.5, and 0.1 mg/mL) were obtained by mixing different concentrations of SOP solution with 5× SDS-PAGE Loading Buffer at a ratio of 4:1 (*v*/*v*) followed by slightly oscillating. The samples were then boiled for 5 min in a palm-sized drive lock incubator (Bio Medical Science Inc., Tokyo, Japan) and briefly centrifuged at 1800× *g* using an S1010E microcentrifuge (SCILOGEX, Rocky Hill, CT, USA). The samples were placed at room temperature, and the Precision Plus protein markers were loaded onto the gel lanes, and then electrophoresis was performed using a Mini-PROTEAN Tetra Cell (Bio-Rad Laboratories Inc., Richmond, CA, USA) at a constant voltage of 180 V for 50 min in 1× Tris/Glycine/SDS buffer. After the gel was discolored in a solution prepared with 0.25% (*w*/*v*) Coomassie Brilliant Blue R250 for 30 min, it was transferred to the decolorizing fluid (Ethanol:Acetic acid:H_2_O = 2:1:7) on a shaker with 80 rpm speed until clear protein bands were observed. Then, the gel was imaged with the Amersham Imager 600 System (Cytiva, Marlborough, MA, USA).

### 4.6. UV–Visible Absorption Spectroscopy of SOP

The ultraviolet absorption spectrum of SOP was measured using a UV spectrophotometer. The SOP was fully dissolved in distilled water to prepare a 0.1 mg/mL sample solution. The sample solution was placed in a 10 mm quartz cuvette for UV scanning. The scanning wavelength range was 190–400 nm, with a scanning speed of 2 nm/s and a scanning interval of 1 nm. Distilled water was used as the blank control.

### 4.7. Amino Acid Composition Analysis

The amino acid content of SOP was analyzed using an LA8080 high-speed automatic amino acid analyzer (Hitachi Koki Co., Ltd., Tokyo, Japan). Briefly, the lyophilized SOP was hydrolyzed in 6 M HCl at 110 °C for 24 h. Excess solvent was evaporated under a vacuum in a desiccator, and the dried sample was redissolved in distilled water. This drying and redissolving process was repeated 3 to 4 times. Finally, the dried sample was dissolved in the minimal volume of sodium citrate buffer (pH 2.2) and filtered through a 0.22 μm nylon filter membrane (Shanghai Titan Technology Co., Ltd., Shanghai, China). The amino acid analyzer was calibrated using standard reagents, with a positive control run for all amino acids prior to analyzing the test sample. The retention times of the amino acid peaks were compared to those of the corresponding positive controls. The content of amino acid is expressed as the number of residues/1000 residues.

### 4.8. Preparation of Protamine Derivatives

The preparation of protamine derivatives was performed following our previous report with slight modifications [71]. Specifically, polyethylene glycol 2000 (PEG) was activated using N-hydroxysuccinimide (NHS). The detailed procedure was as follows: PEG was dissolved in anhydrous DMSO, followed by the addition of NHS and 1-ethyl-3-(3-dimethylaminopropyl) carbodiimide (EDC). The mixture was stirred at room temperature for 2 h to activate PEG. Subsequently, the activated PEG was added to the SOP solution at a 1:1 (M/M) ratio and stirred with a magnetic stirrer at room temperature for 24 h to facilitate covalent conjugation between PEG and SOP. After the reaction, the solution was dialyzed against distilled water using a dialysis membrane (MWCO 3.5 kDa) for 48 h to remove unreacted PEG and small molecular impurities. The dialyzed solution was then freeze-dried using a freeze dryer (7420070, Labconco, Kansas City, MO, USA) to obtain the SOP-PEG conjugate.

### 4.9. Fourier Transform Infrared (FTIR) Analysis of SOP and SOP-PEG

The FTIR spectra of SOP and SOP-PEG were determined using a Fourier transform infrared spectrometer (L1050050 Spotlight 400, PerkinElmer Co., Wellesley, MA, USA). The spectra were collected using air as the background to eliminate potential interferences. The scanning range was set between 1000 and 4000 cm^−1^, with a resolution of 4 cm^−1^, and each sample was scanned 32 times. The secondary structure of SOP was further analyzed in the amide I region (1600–1700 cm^−1^) using PeakFit Version 4.12 software (SeaSolve Software Inc., Framingham, MA, USA). The percentage of each secondary structure was calculated by dividing the peak area of each individual structure by the total peak area of all secondary structures.

### 4.10. Circular Dichroism (CD) Spectrum

The CD spectra of SOP and SOP-PEG were measured using a BRIGHTTIME Chirascan spectropolarimeter (Applied Photophysics Ltd., Surrey, UK). Lyophilized SOP and SOP-PEG samples were fully dissolved in distilled water to ensure complete solubilization. The sample solutions were then transferred into a quartz cuvette with a 1 mm path length. CD spectra were recorded at 25 °C over a wavelength range of 190–300 nm, with a scanning speed of 100 nm/min. Distilled water was used as the blank control.

### 4.11. Microstructural Analysis of SOP and SOP-PEG

The changes in the structural properties between SOP and SOP-PEG were determined by scanning electron microscopy (SEM). The freeze-dried protamine samples were adhered to aluminum stubs with conductive adhesive tape, and aligned in the desired orientation. The samples were then placed in an ion sputter coater for gold deposition to enhance conductivity. The metalized collagen samples were mounted onto SEM sample holders (20 s glow discharge carbon-supported adhesive films) and subsequently transferred to the sample chamber for analysis. The surface morphology of SOP and SOP-PEG was observed using a SU5000 thermal field emission scanning electron microscopy (Hitachi Koki Co., Ltd., Tokyo, Japan) at an accelerating voltage of 3 kV.

### 4.12. TGA

The thermal stability of SOP and SOP-PEG was evaluated using a NETZSCH TG 209 F1 LIBRA thermogravimetric analyzer (Selb, Germany). Approximately 5 mg of each sample was placed evenly in an alumina crucible and exposed to a nitrogen atmosphere (purity ≥ 99.9%). The samples were heated from 20 °C to 500 °C at a rate of 10 °C/min. An empty aluminum pan was used as a reference. The derivative thermogravimetric (DTG) curve was obtained from the differential thermogravimetric (DTG) data

### 4.13. Culture of L-929 Cells

L-929 cells were obtained from the China Center for Type Culture Collection (CCCTC), Chinese Academy of Sciences (Shanghai, China). The cells were cultured in DMEM (Gibco, Thermo Fisher Scientific, Waltham, MA, USA), supplemented with 10% FBS (Gibco, Thermo Fisher Scientific, Waltham, MA, USA) and 1% P/S, in an incubator at 37 °C with 5% CO_2_.

### 4.14. Cell Viability Assay

Cell proliferation was assessed using a Cell Counting Kit-8 (CCK-8) assay (AbMole, Houston, TX, USA) according to the manufacturer’s instructions. L-929 cells (1 × 10^4^ cells/well) were seeded in a 96-well plate and cultured until approximately 80% confluence. Different concentrations of SOP, SOP-PEG, or the control (PBS) were added to each well, and the cells were further incubated for 48 h. Following the manufacturer’s protocol, 10 μL of CCK-8 reagent was added to each well and incubated for 1 h. Absorbance (OD value) was measured at 450 nm using a microplate reader (BioTek, Winooski, VT, USA).

### 4.15. Cell Cycle Assay

Cell cycle analysis was performed using a PI Cell Cycle and Apoptosis Analysis Kit (Beyotime, Shanghai, China). L-929 cells (1 × 10^5^ cells/well) were seeded in a 6-well plate and treated with different concentrations of SOP or SOP-PEG for 24 h. After treatment, cells were collected and fixed in 70% cold ethanol and stored at 4 °C overnight. The cells were then stained with PI dye and incubated at room temperature for 30 min. Apoptosis was determined using BD FACS Celesta flow cytometry (BD Biosciences, Franklin Lakes, NJ, USA). The distribution of cells in the G0/G1, S, and G2/M phases was analyzed using FlowJo V10 software.

### 4.16. Quantitative Real-Time Polymerase Chain Reaction (qRT-PCR)

After 24 h of treatment with different concentrations of SOP or SOP-PEG, total RNA was extracted from L-929 cells using the SteadyPure Quick RNA Extraction Kit (Accurate, Changsha, China) according to the manufacturer’s protocol. After DNase treatment, the RNA was reverse-transcribed into single-stranded cDNA using the cDNA First Strand Synthesis Kit. Quantitative RT-PCR was performed using the SYBR Green Premix Pro Taq HS qPCR Kit, with gene-specific primers, in a 20 µL reaction system. The PCR cycling conditions were as follows: an initial denaturation at 95 °C for 30 s, followed by denaturation at 95 °C for 5 s and annealing at 60 °C for 30 s, for a total of 40 cycles. The specific primers were synthesized by Sangon Biotech, with sequences listed in Table 3. GAPDH was used as the internal control. The mRNA expression levels of target genes were calculated using the comparative CT method.

### 4.17. Western Blot Analysis

Cells were lysed on ice for 20 min using RIPA lysis buffer containing protease inhibitors, and the supernatant was collected after centrifugation at 4 °C. Protein concentration was determined using a BCA Protein Assay Kit (TIANGEN, Shanghai, China). Equal amounts of total protein were resuspended in the sample buffer and heated at 100 °C for 3 min. The proteins were separated by 7.5–15% SDS-PAGE and then transferred to a PVDF membrane (Merck Millipore, Darmstadt, Germany). After blocking with 5% non-fat milk, the membrane was incubated overnight at 4 °C with the primary antibody (appropriately diluted). Following three washes with TBST, the membrane was incubated with HRP-conjugated secondary antibody at room temperature for 1 h. After washing, protein bands were visualized using enhanced chemiluminescence (ECL) reagents and captured with an Amersham Imager 600 system. Quantification of protein bands was performed using ImageJ software (version 1.54p).

### 4.18. Statistical Analysis

Data are expressed as the mean ± standard deviation of three replicates. Significant differences between the means of parameters were determined using Duncan’s multiple-range test and one-way analysis of variance (ANOVA) with SPSS 17.0 (SPSS, Inc., Chicago, IL, USA). A *p*-value of <0.05 was considered statistically significant.

## 5. Conclusions

This research presents, for the first time, the biomedical potential of protamine (SOP) extracted from the mature spermatogonial tissues of *S. oualaniensis*, along with its PEGylated form (SOP-PEG). The process of PEGylation significantly improved the stability and bioactivity of SOP, particularly in enhancing cell proliferation, regulating the cell cycle, and boosting anti-apoptotic properties. Additionally, PEGylation increased the delivery efficiency of SOP, underscoring its potential applications in skin repair and biopharmaceuticals. Importantly, the modification with PEG greatly enhanced the biological functions of SOP, making it a promising candidate for regenerative medicine. SOP-PEG, in particular, exhibits better biocompatibility and efficacy, supporting its role as a therapeutic carrier for wound healing and regenerative medicine. Moreover, its application in biopharmaceuticals, especially in the delivery of protein drugs, highlights its clinical potential. Nevertheless, despite the encouraging advancements brought about by PEGylation, challenges remain for clinical application, including the effects of PEG molecular weight and the degree of modification on therapeutic effectiveness, as well as potential immunogenicity concerns that may restrict long-term use. Consequently, optimizing PEGylation techniques, assessing therapeutic outcomes, and addressing immunogenic issues will be essential areas for future investigation. Overall, this study offers important insights into the potential uses of SOP and its PEGylated derivative in regenerative medicine while also pinpointing challenges for clinical implementation.

## Figures and Tables

**Table 1:** Amino acid composition of sepia sperm proteins compared to other fish species’ sperm proteins (residues/1000 residues).

Amino Acid Species	SOP	Takifugu Flavidus [39]
Aspartic acid (Asp)	41.3 ± 0.9	68.4
Threonine (Thr)	38.5 ± 0.4	48.4
Serine (Ser)	55.9 ± 0.2	66.1
Glutamic acid (Glu)	39.3 ± 0.6	52.3
Glycine (Gly)	41.8 ± 0.4	67.2
Alanine (Ala)	46.8 ± 0.3	173.9
Cysteine (Cys)	16.2 ± 0.1	-
Valine (Val)	30.8 ± 0.2	58.6
Isoleucine (Ile)	47.2 ± 0.3	48.9
Leucine (Leu)	60.3 ± 0.2	62.7
Tyrosine (Tyr)	41.2 ± 0.1	54.7
Phenylalanine (Phe)	30.5 ± 0.1	43.8
Lysine (Lys)	106.8 ± 0.3	-
Histidine (His)	20.6 ± 0.02	20.5
Arginine (Arg)	290.7 ± 0.1	199.2
Proline (Pro)	92.1 ± 0.4	35.3
Total amino acid	1000	1000
Basic amino acid	418.1 ± 0.3	219.7

Note: “-” indicates values below the detection limit (<0.002), not detected.

**Table 2:** According to the CD results of SOP and SOP-PEG, the DichroWeb site was used to calculate the secondary structure content [47].

	α-Helix	β-Sheet	β-Turn	Random
SOP	21.7%	0%	26.5%	51.8%
SOP-PEG	53.1%	46.9%	0%	0%

**Table 3:** Primer sequences in RT-PCR used in the measurement of mRNA expression.

Gene	Primer Sequence
*CDK1*	Forward:5′-CTGTTTGCTGATCTGGAGCTG-3′
	Reverse: 5′-GTCCAGGGATTCTTCAGTGG-3′
*PLK1*	Forward: 5′-ATGTTGCACCGTTCTGTCAC-3′
	Reverse: 5′-GCTCCTCATGTTGTCGTAGC-3′
*PI3K*	Forward: 5′-TCTCGGCCTGCTCTACATTC-3′
	Reverse: 5′-GCTGCCGTACTTGGTCACTC-3′
*Akt*	Forward: 5′-TGACGACGTGGCTATTGTGA-3′
	Reverse: 5′-TCCATTGAGGTGCCTGTCAT-3′
*Bax*	Forward: 5′-GTTTCATCCAGGATCGAGCAG-3′
	Reverse: 5′-GAAAGTAGAAAAGGGCGACAAC-3′
*Bcl-2*	Forward: 5′-GTCGCTACCGTCGTGACTTC-3′
	Reverse: 5′-CAGACATGCACCTACCCAGC-3′
*GAPDH*	Forward: 5′-GGTTGTCTCCTGCGACTTCA-3′
	Reverse: 5′-TGGTCCAGGGTTTCTTACTCC-3′

## Data Availability

Data are contained within the article.

## Supplementary Materials

The following supporting information can be downloaded at https://www.mdpi.com/article/10.3390/ijms26051869/s1.
