# Supplementary material for: Polyethylene Glycolylation of the Purified Basic Protein (Protamine) of Squid (Symplectoteuthis oualaniensis): Structural Changes and Evaluation of Proliferative Effects on Fibroblast
Source: Int J Mol Sci. 2025 Feb 21;26(5):1869. doi: 10.3390/ijms26051869 (PMC11899872; doi:10.3390/ijms26051869)

Preparation of Protamine Derivatives

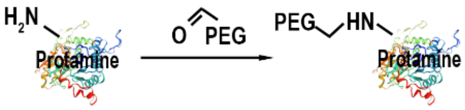

Figure S1. Western blot for Figure 2a

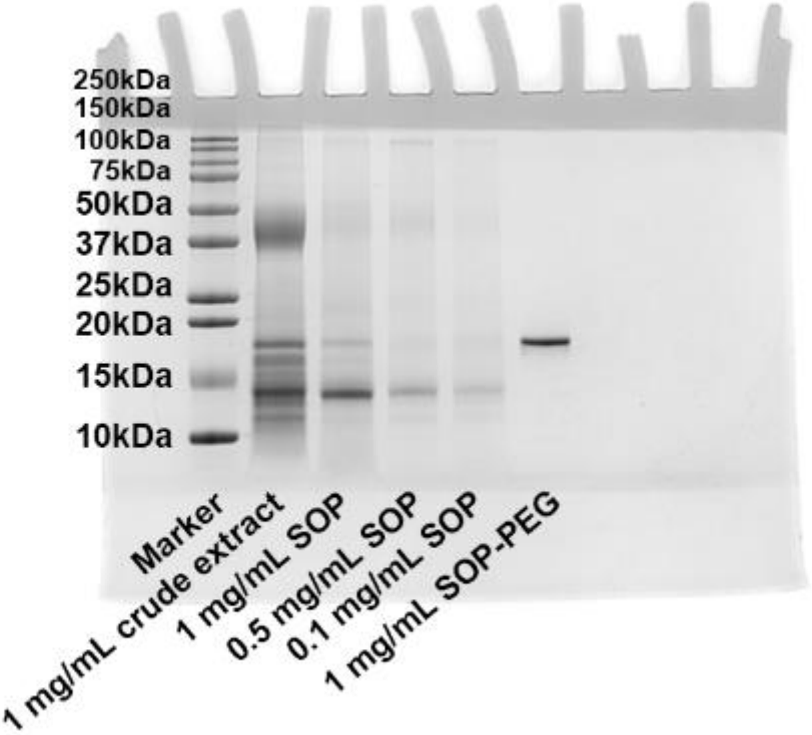

Figure S2. Western blot for Figure 6b

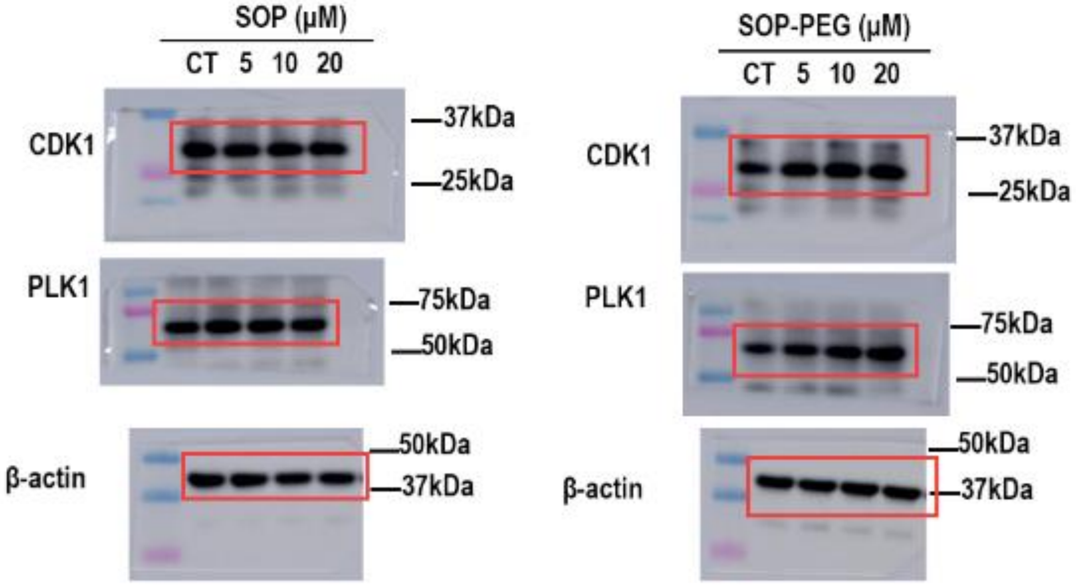

Figure S3. Western blot for Figure 7b

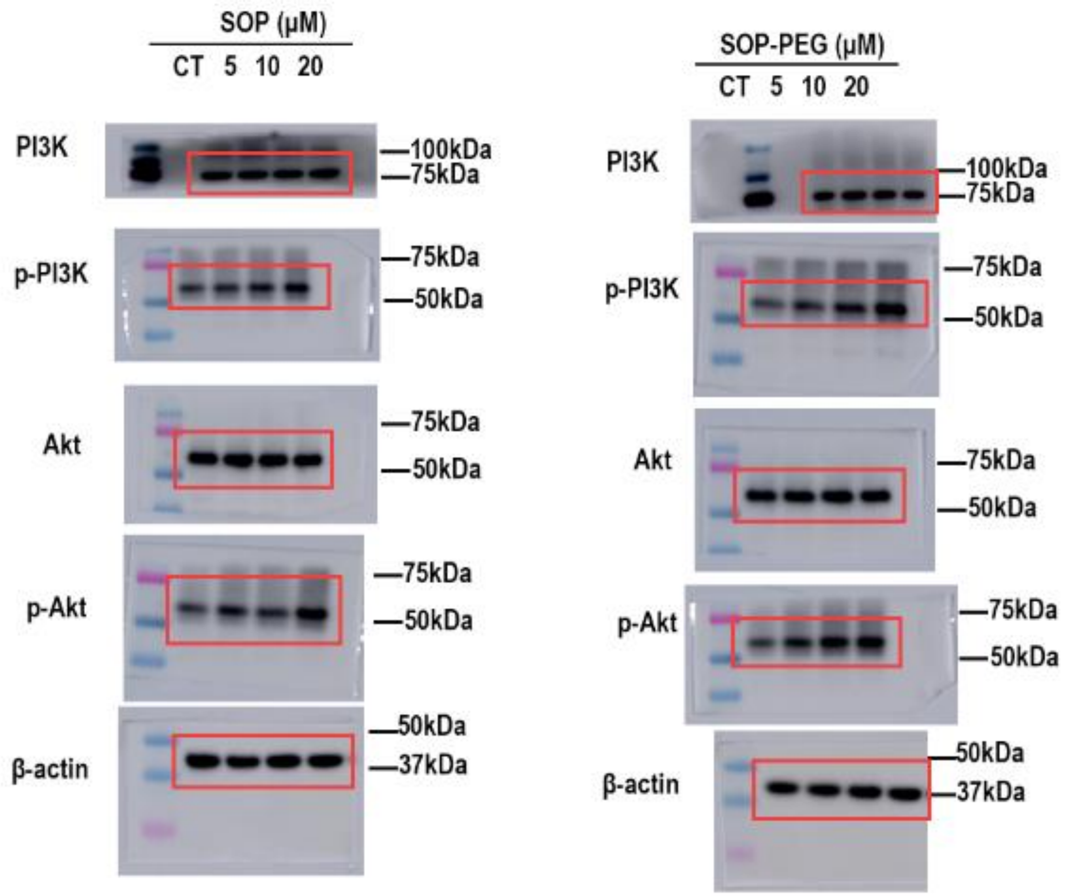

Figure S4. Western blot for Figure 8b

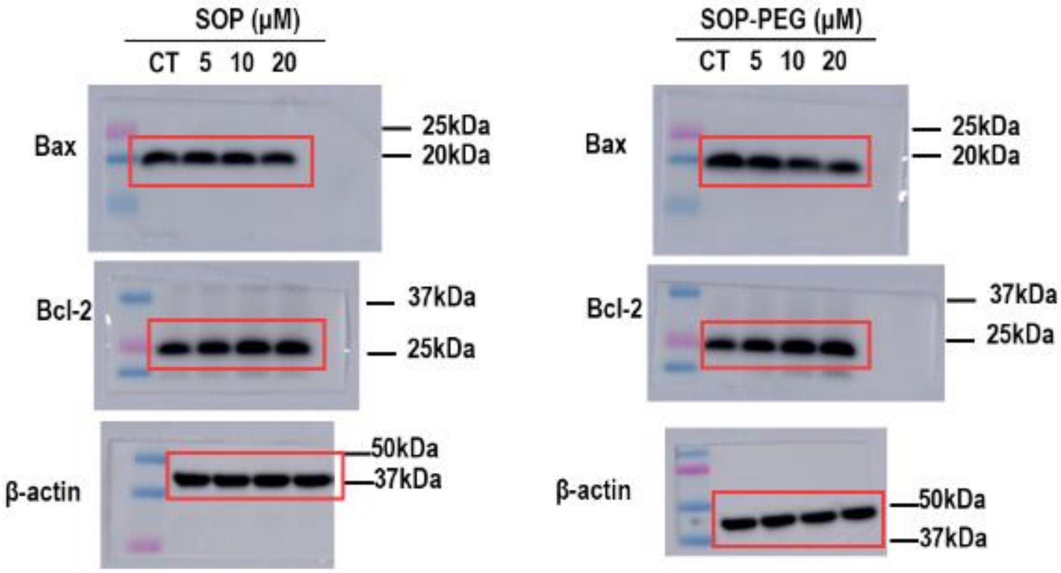

Supplement: Supplementary file 1 [file ijms-26-01869-s001.zip › ijms-3447529-supplementary.pdf]
